# Functional gene pyrosequencing and network analysis: an approach to examine the response of denitrifying bacteria to increased nitrogen supply in salt marsh sediments

**DOI:** 10.3389/fmicb.2013.00342

**Published:** 2013-11-27

**Authors:** Jennifer L. Bowen, Jarrett E. K. Byrnes, David Weisman, Cory Colaneri

**Affiliations:** Department of Biology, University of Massachusetts BostonBoston, MA, USA

**Keywords:** DNA sequence clustering, AIC, network analysis, *nirS*, salt marsh, eutrophication, functional gene pyrosequencing

## Abstract

Functional gene pyrosequencing is emerging as a useful tool to examine the diversity and abundance of microbes that facilitate key biogeochemical processes. One such process, denitrification, is of particular importance because it converts fixed nitrate (NO^−^_3_) to N_2_ gas, which returns to the atmosphere. In nitrogen limited salt marshes, removal of NO^−^_3_ prior to entering adjacent waters helps prevent eutrophication. Understanding the dynamics of salt marsh microbial denitrification is thus imperative for the maintenance of healthy coastal ecosystems. We used pyrosequencing of the *nirS* gene to examine the denitrifying community response to fertilization in experimentally enriched marsh plots. A key challenge in the analysis of sequence data derived from pyrosequencing is understanding whether small differences in gene sequences are ecologically meaningful. We applied a novel approach from information theory to determine that the optimal similarity level for clustering DNA sequences into OTUs, while still capturing the ecological complexity of the system, was 88%. With this clustering, phylogenetic analysis yielded 6 dominant clades of denitrifiers, the largest of which, accounting for more than half of all the sequences collected, had no close cultured representatives. Of the 638 OTUs identified, only 11 were present in all plots and no single OTU was dominant. We did, however, find a large number of specialist OTUs that were present only in a single plot. The high degree of endemic OTUs, while accounting for a large proportion of the *nirS* diversity in the plots, were found in lower abundance than the generalist taxa. The proportion of specialist taxa increased with increasing supply of nutrients, suggesting that addition of fertilizer may create conditions that expand the niche space for denitrifying organisms and may enhance the genetic capacity for denitrification.

## Introduction

Salt marshes, located along the shores of temperate coastal waters around the world, provide more ecosystem services than any other coastal habitat (Gedan et al., 2009). These services include shoreline stabilization, nursery and breeding grounds for commercially important finfish and shellfish species, and interception of land-derived contaminants (Valiela et al., 2004). Salt marshes also sequester more carbon in their soils than any other temperate biome (Duarte et al., 2005) and contribute 1% to the global loss of fixed nitrogen through microbially-mediated denitrification (Seitzinger et al., 2006). In 2007 the value of the ecosystem services provided by salt marshes was estimated at $14,397 ha^−1^ y^−1^, of which 66% was attributed to services associated with nutrient removal and transformation (Gedan et al., 2009), much of which occurs as a result of microbial metabolisms. It is clear that the estimated 40 million hectares of salt marsh area in the world (Duarte et al., 2005) are teeming with microbes that provide considerable benefit to human kind, yet we know little of the diversity and function of the microbes responsible for these critical ecosystem services.

Despite their economic importance, salt marshes are under considerable threat from anthropogenic causes (Gedan et al., 2009; Deegan et al., 2012). Located at the interface between land and sea, marshes are subject to perturbations from both biomes. Increasing pressure from expansion of human activities in the coastal zone has resulted in increased delivery of land-derived nutrients to estuarine habitats (Valiela et al., 1992; Howarth et al., 2002; Cole et al., 2006). Since nitrogen (N) is the nutrient typically limiting primary production in salt marshes (Valiela and Teal, 1979) and estuaries (Vitousek and Howarth, 1991; Paerl, 1997), excess anthropogenic nitrogen additions have resulted in a cascade of deleterious effects in both coastal waters and their associated wetlands (Bertness et al., 2002; Diaz and Rosenberg, 2008; Turner et al., 2009; Deegan et al., 2012). Concurrent with increasing nutrient enrichment, rising sea levels can result in the landward movement of salt marshes. In many regions human modification of shorelines prevents such landward migration, which could ultimately result in drowning and loss of marsh area (Donnelly and Bertness, 2001). Losses of wetland area from changing land use on shorelines, shifting hydrologic baselines, and nutrient enrichment have also been well-documented (Bertness et al., 2002; Deegan et al., 2012) and constrain the capacity of salt marshes to provide the ecosystem services upon which society depends.

The anthropogenic threat to salt marshes, coupled with the economic importance of these habitats, has motivated researchers to undertake extensive studies on the extent to which these wetlands can remove anthropogenic nitrogen. Early recognition of salt marshes' role as a sink for land-derived nitrogen led to the establishment of different experimental systems that examine how nutrient enrichment alters marsh macroecology and biogeochemistry (Valiela et al., 1975; Deegan et al., 2007). Multiple studies indicate that increasing anthropogenic N alters rates of N loss processes including denitrification, coupled nitrification-denitrification, and dissimilatory nitrate reduction to ammonia (DNRA) (Hamersley and Howes, 2005; Koop-Jakobsen and Giblin, 2010; Vieillard and Fulweiler, 2012; Kinney and Valiela, 2013). It is only within recent years, however, that genetic tools have been developed to examine, in detail, the microbial communities that underlie the biogeochemistry of marsh systems. Recent terminal restriction fragment length polymorphism analysis of the ammonia monooxygenase gene in salt marsh plots exposed to sewage sludge suggests that increased nutrient supply may shift the community structure of ammonia oxidizing bacteria but not ammonia oxidizing archaea (Peng et al., 2012). Lage et al. (2010) also show a response by ammonia oxidizing bacteria to nitrogen enrichment, but nitrogen plus phosphorus additions show no response among the ammonia oxidizing bacteria. Using denaturing gradient gel electrophoresis, Piceno and Lovell (2000) also showed little change to the nitrogen fixing microbial community as a result of short-term nutrient additions. Functional gene microarray analysis of the *nirS* gene, a key gene in the denitrification pathway, also showed little change in the denitrifying community as a result of N addition, at least among the subset of denitrifiers detected on the microarray chip (Bowen et al., 2011).

In this study we use functional gene pyrosequencing of the *nirS* gene to explore the diversity and structure of the *nirS* gene-containing members of the denitrifying community in salt marsh sediments exposed to different degrees of nutrient enrichment. A key challenge in the analysis of the massive amounts of sequence data derived from functional gene pyrosequencing comes in understanding whether small differences in gene sequences are ecologically meaningful. Here we use an information theory approach modified from food web network analysis (Allesina and Pascual, 2009) to ascertain the most parsimonious degree of sequence clustering that reduces taxonomic complexity without sacrificing significant ecological information loss. Specifically, we derive a network between the representative cluster sequence and its abundance in each marsh plot to calculate the Akaike Information Criteron (AIC) score for each clustering. The AIC score indicates the degree of network complexity and fine-scale ecological information about individual sequences that gets lost as sequence clustering increases. In essence, our clustering methodology ends up creating the most parsimonious OTU assignments based jointly on taxonomic and ecological similarity of individual sequences, balancing the conflicting goals of using generalized OTU clustering (e.g., 97% sequence similarity used for 16S rRNA studies) vs. clustering that is specific to this system alone.

## Materials and methods

### Study site and sample collection

**Figure 1:**
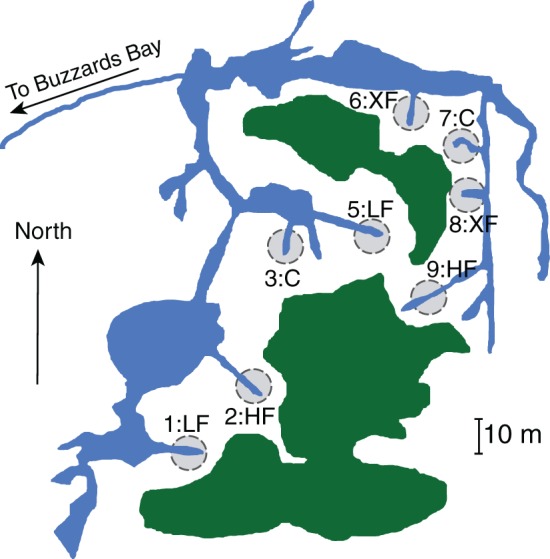
**Location and treatment level for each of the marsh plots at Great Sippewissett Marsh, Falmouth, MA, USA**. Fertilizers were added in the following doses: Control (C): no fertilizer added, low dose (LF): 0.85 g N m^‒2^ wk^‒1^, high dose (HF): 2.52 g N m^‒2^ wk^‒1^, and extra high dose (XF) 7.56 g N m^‒2^ wk^‒1^. Figure modified from Kinney and Valiela (2013).

We collected samples from the salt marsh fertilization plots at Great Sippewissett Marsh, Falmouth, MA, in September 2009. The long-running Sippewissett Marsh experiment has been described elsewhere (Valiela et al., 1975, 1976) but briefly, the marsh fertilization experiment began in the early 1970s. Each plot is 10 meters in diameter and each bisects a marsh creek terminus so that all marsh habitats (creek bank, low marsh, high marsh) are equivalently represented in each of the plots (Figure 1). Duplicate plots were randomly assigned to treatments and have been fertilized (via addition of pelletized sewage sludge) biweekly through the growing season at the following rates: Control (C): no fertilizer added, low dose (LF): 0.85 g N m^−2^ wk^−1^, high dose (HF): 2.52 g N m^−2^ wk^−1^ and extremely high dose (XF): 7.56 g N m^−2^ wk^−1^. We collected surface sediments (top 1 cm, encompassing the entire redox gradient) using a sterile 25 cc syringe corer. Duplicate cores from each plot were homogenized and duplicate subsamples were immediately frozen in liquid nitrogen and returned to the laboratory where they were stored at −80°C until DNA extraction.

Cores in each plot were collected from the rooting zone of pure or nearly pure stands of the tall ecotype of *Spartina alterniflora*, taking care to avoid the edge of the plots that are in close contact with the creek banks. We restricted our sampling to a single vegetation type to avoid differences in microbial community structure that might result from sampling the rooting zone of different plant taxa. Thus, we do not intend that the microbial assemblages that we document here are representative of entire salt marsh plots. Rather, we are specifically testing how the microbial community in one salt marsh habitat responds to increased supplies of anthropogenic nitrogen.

### Dna extraction, amplification, and sequencing

Environmental DNA from the eight salt marsh plots were extracted in triplicate using the PowerSoil^®^ DNA Isolation Kit from MoBio Laboratories (Carlsbad, CA). DNA products were pooled and amplified with primers nirS1F and nirS6R (Braker et al., 1998), in three independent PCR runs. The PCR reaction consisted of a 1:10 dilution of template DNA, and final concentrations of 1× Phusion HF Buffer, 1.5 mM MgCl_2_, 1.6 mM dNTP, 0.4 μM forward and reverse primers, 400 μg/mL BSA, 3% DMSO, and 0.02 U/μL Phusion DNA polymerase (New England Biolabs, Ipswich, MA). Reaction conditions included denaturation for 2 min at 98°C, 29 cycles of 98°C for 10 s, 61°C for 30 s, and 72°C for 1 min, followed by a final extension step at 72°C for 5 min. PCR products were agarose gel verified and pooled. Nested PCR was performed on 0.15% (v/v in final PCR reaction mixture) of the pooled PCR products using pyrosequencing adapted nirS3F and nirS6R primers in three independent PCR runs for each sample. The reaction conditions were the same as above, but with 1.75 mM MgCl_2_, 0.1 μ M forward and reverse primers, and an annealing temperature of 56°C. Gel purified PCR amplicons were quantified via Quant-IT™ Picogreen^®^ Reagent from Invitrogen (now Life Technologies, Grand Island, NY) on an Agilent MX3005p qPCR system (Santa Clara, CA). Samples were eluted in 10 mM Tris-HCl buffer (pH 8) to a concentration of 10 ng μl^−1^ and combined in equal ratios for pyrosequencing on Roche's 454 FLX Genome Sequencer (Branford, CT) at the Josephine Bay Paul Center for Comparative Molecular Biology and Genomics at the Marine Biological Laboratory in Woods Hole, MA. Pyrosequencing was performed using the 454 Titanium sequencing technology following manufacturers instructions.

### Processing pyrosequencing data

Following pyrosequencing, sequences with mismatches to the 5′ primer were removed, as were sequences containing any ambiguous bases. The remaining sequences were trimmed to 432 bp and were processed using FunFrame (Weisman et al., 2013), a functional gene analysis pipeline we developed for the high throughput analysis of protein coding gene amplicons. Briefly, FunFrame uses HMM-FRAME (Zhang and Sun, 2011) along with a hidden Markov model of the cytochome D1 *nirS* gene from Pfam (accession PF02239.10) to identify and correct frameshift errors that result from homopolymer misreads. The pipeline also examines sequences for chimeras using UCHIME run in *de novo* mode (Edgar et al., 2011). ESPRIT-Tree (version 11152011; Cai and Sun, 2011) is embedded in FunFrame to cluster sequences into operational taxonomic units (OTUs) and is parameterized by the sequence similarity within OTU clusters. For the analyses reported here, we extended FunFrame to iterate this parameter over a range of sequence similarities, from 74 to 99%, to derive a series of OTU clusterings (Table S1). We used each cluster, along with the network generated between the representative cluster sequence and its abundance in each of the salt marsh plots, to calculate the AIC score for each cluster. The AIC score provides an assessment of the degree of complexity lost with each step increase in sequence clustering, thereby providing a guideline for determining the degree of clustering that is ecologically relevant.

We used the percent sequence similarity with the lowest AIC score to cluster our sequences using ESPRIT-Tree. A representative sequence from each cluster, along with a number of reference sequences derived from sequenced genomes containing the *nirS* gene (Jones and Hallin, 2010) were aligned using PyNAST (Caporaso et al., 2010). The alignment was used as input to FastTree (Price et al., 2010) to generate a phylogenetic tree. This tree was visualized using the Interactive Tree of Life (Letunic and Bork, 2011). Representative sequences from the most abundant clades were assigned taxonomy using a BLASTn search against the NCBI nucleotide collection. All sequence data were submitted to NCBI's Sequence Read Archive (Accession number SRP029151).

### Calculating AIC

We calculated AIC scores by modifying an approach pioneered in food web network analysis (Allesina and Pascual, 2009) to determine the largest degree of sequence aggregation we could perform without sacrificing too much ecologically relevant complexity in our model. To do this, we took advantage of the fact that we can define a bipartite network *N*(*S* + *r*, *L*) with *S* + *r* nodes consisting of *S* sequence types connecting to *r* plots by *L* links. These sequences are grouped into *k* OTUs. We first worked with a binary network (all edges have equal weight) where an OTU and plot are considered connected when a sequence from an OTU is found in a plot. Defining the probability of choosing any sequence from OTU *i* at random and finding a link to plot *q* with *L*_*iq*_ links of sequences in OTU *i* to plot is,

(1) $$p(iq)=L_{iq}/S_{i}$$

where *S*_*i*_ is the number of sequence types in an OTU. We can modify the equations from Allesina and Pascual (2009) to calculate the probability of any given OTU-plot association in network structure *N*, given the observed *p*(*iq*), such that,

(2) $$P(N|p(iq))=p{\left(iq\right)}^{Liq}{\left(1-p(iq)\right)}^{Liq-Si}$$

This translates to a log likelihood of any given OTU-plot association given network *N* as,

(3) $$LL\;(iq|N)=L_{iq}\log(p(iq))+\;(L_{iq}-S_{i})\log(1-p(iq))$$

Summing over all OTU-plot associations gives a log-likelihood value for the network as a whole. In any given network configuration with *S* sequences, *k* groups, and *r* sites, there are *2S* + *2kr* parameters for the total log-likelihood calculation. Thus, taking 0log(0) = 0, the AIC value for an imposed network configuration is,

(4) $$\text{AIC​}\left(N\right)=2kr+2S-2\sum_{i=1}^{k}\sum_{q=1}^{r}LL(iq|N)$$

Thus, we are able to calculate the AIC of the structures implied by different OTU configurations based on 74% sequence similarity to 99% sequence similarity. Minimizing the AIC yields the OTU structure that best reproduces the observed patterns of sequences in the data while minimizing the complexity of our OTU structure.

We know, however, that sequences vary in abundance across plots. We want to define an “optimal” OTU structure as one where species within an OTU not only exhibit similar patterns of co-occurrence, but also similar abundances when they co-occur. To incorporate abundance information into our network AIC calculations, we took our bipartite network and assigned a weight to each edge based on sequence abundance. Using this weighted network, we calculated the log-likelihood of observing the distribution of abundances of sequences in OTU *i* in plot *q*. We began by assuming that observed sequences were Poisson distributed within a plot. Defining *a*_*jiq*_ as the abundance of sequence *j* in group *i* and plot *q*, *a*_*.iq*_ as the summed abundance of all sequences in group *i* and plot *q*, we can calculate the log-likelihood of observing a pattern of abundances *A* of sequence types in group *i* in plot *q* as,

(5) $$LL\text{​}\left(iq|A\right)=\sum_{i=1}^{j}\text{Poisson}\text{​}\left(a_{jiq};\lambda =\frac{a_{.iq}}{S_{i}}\right)$$

We also considered using a Binomial distribution instead of a Poisson so that,

(6) $$LL\text{​}\left(iq|A\right)=\sum_{i=1}^{j}\text{Binomial}\text{​}\left(a_{jiq};\text{size}=a_{.iq},p=\frac{1}{S_{i}}\right)$$

But we found little difference (optimal clustering at 92% instead of 88%), and so do not report the results here. Adding abundances adds 2*kr* more parameters, so, now, an OTU-site network AIC is as follows,

(7) $$\text{AIC​}\left(N\right)=4kr+2S-2\sum_{i=1}^{k}\sum_{q=1}^{r}\left[LL(iq|N)+LL(iq|A)\right]$$

Again, the lowest AIC score indicates the degree of clustering that provides the best descriptor of the network structure while penalizing for an overly complex description of the system. All analyses were carried out in R 2.15.2 (R Core Team, 2013).

### Network visualization

After determining the optimal OTU-plot network structure, we examined the resulting OTU-plot network in several ways. First, we plotted bipartite OTU-plot networks at different levels of sequence clustering with edge width proportional to abundance of an OTU in a given plot. Second, we plotted the network of OTUs and salt marsh plots as a graph with marsh plots included as their own nodes using the Fruchterman and Reingold (1991) algorithm for ease of visualization. We then investigated (1) the distribution of the number of marsh plots in which each OTU occurred (i.e., from specialists occurring in one plot to generalists occurring in all eight), and (2) the distribution of abundances of OTUs and their pattern of plot specialization. Plots were made using *ggplot2* (Wickham, 2009) and *network* (Butts et al., 2012) libraries. Frequency of co-occurrence was calculated as the fraction of OTUs shared between two plots. The heatmap was visualized using the heatmap2 function in the *gplots* library (Warnes et al., 2012). Clustering for the dendrogram was determined using the hclust function in *gplots*, which performs complete linkage hierarchical clustering.

## Results

### Determining ecologically relevant sequence clustering of the *nirS* gene

Pyrosequencing of functional genes has the capacity to produce a tremendous amount of sequence data on the distribution and abundance of specific protein coding genes in the environment. The eight salt marsh samples analyzed in this study represent only a subset of the samples pooled into one pyrosequencing run. After low quality sequences were removed, we sequenced between 2122 and 4733 different OTUs of the *nirS* genes per marsh plot derived from a grand total of 66,279 sequences that were retained. With such tremendous sequence diversity it becomes challenging to determine at what point two orthologous genes that have somewhat divergent sequences are ecologically distinct from one another.

To illustrate this challenge, we first clustered all the *nirS* sequences from the marsh plots at a sequence similarity of 99% (Figure S1A) and then clustered the same sequences at a sequence similarity of 76% (Figure S1B). When OTUs were defined at greater than 99% sequence similarity (Figure S1A) there were 7790 different sequences among all the plots, of which between 473 and 1258 sequences were present as singletons (occurring only one time in only one plot). By contrast, an OTU definition at 76% sequence similarity (Figure S1B) yielded 27 OTUs, of which only one was present in all the plots and six were present only one time. Somewhere in between these two extremes lies a degree of sequence clustering that reduces the overall sequence complexity without sacrificing ecological relevance.

**Figure 2:**
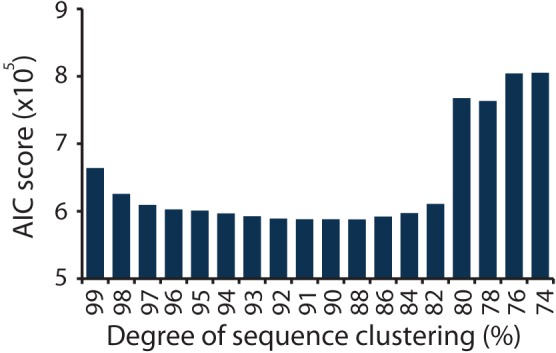
**AIC scores calculated from the bipartite network of sequences and plots, for sequences clustered at a range of similarities, from 74 to 99%**.

In this study the *nirS* DNA sequences derive from eight ecologically meaningful units in the form of duplicated salt marsh plots that have been exposed to specific degrees of nutrient enrichment for over 40 years. Using the network formed between the marsh plots and the *nirS* sequence information, we calculated AIC scores for each degree of clustering (Figure 2). The lowest AIC score was achieved at a clustering of 88% sequence similarity (Table S2). Ninety and eighty-six percent clusters resulted in AIC values that were >250 larger (Table S2). As a result of the AIC analysis we use a clustering of 88% sequence similarity to define OTUs for downstream ecological analysis.

### Community structure of *nirS* genes in fertilized salt marsh plots

When clustered at 88% sequence similarity, we reduced the number of different OTUs from 7790 to 638 (Figure S2, Table 1). Each plot contained between 86 and 265 different OTUs (Table 1), of which between 16 and 78 were singletons. Although there was no significant difference between the numbers of different sequences in the plots that receive higher doses of nitrogen (plots 2, 6, 8, and 9) compared to the low dose and control plots (plots 1, 3, 5, and 7; Student's *t*-test, *p* = 0.099), there were more singletons in the high dose plots relative to the low dose and control plots (Student's *t*-test, *p* = 0.047).

**Table 1:** **Number of total sequences, total number of unique OTUs, and number of plot endemic OTUs, along with the number of singletons, the number of generalist sequences, and the number of endemic sequences found in each of the salt marsh plots when clustered at 88% sequence similarity**.

	**Control**		**Low fert**		**High fert**		**Extra fert**	
Plot number	3	7	1	5	2	9	6	8
Total number of sequences	8281	8588	8023	9729	4440	12,244	10,693	5231
Number of unique OTUs	106	128	86	141	195	158	265	112
Number of plot endemic OTUs	21	29	23	30	70	45	140	32
Number of singletons	18	16	15	23	51	32	78	27
Number of generalist^1^ sequences	7974	8157	7660	8901	2857	10420	4310	532
Number of endemic sequences	25	55	58	39	164	81	660	113

1 Number of generalists defined as the total number of sequences from OTUs found in at least six of the eight plots.

**Figure 3:**
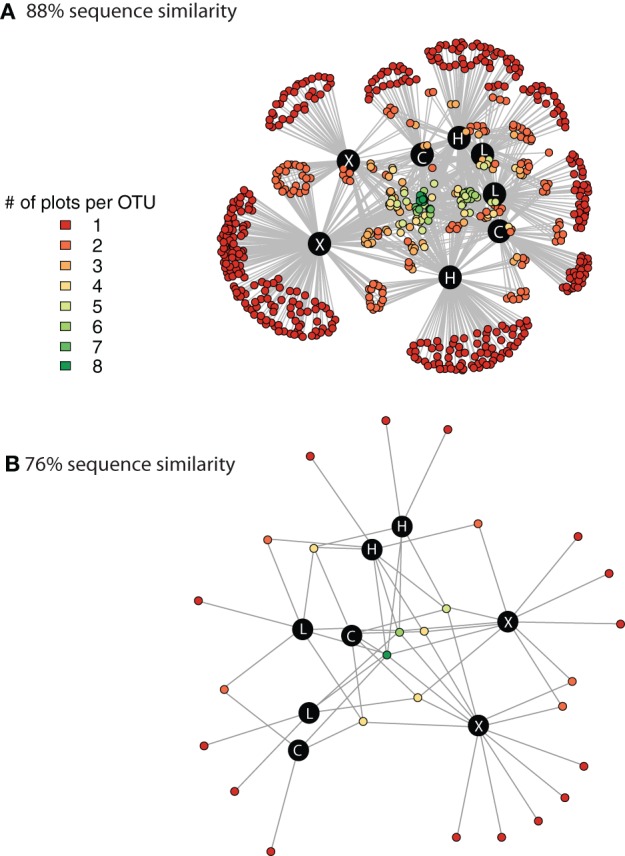
**Network graph displaying the connectivity among marsh plots derived from clustering at 88% sequence similarity (A) and at 76% sequence similarity (B)**. The different colors indicate the number of different plots that contain each individual OTU. The black circles denote the 8 marsh plots and the white lettering indicates the treatment level of the plot. C, control; L, Low dose; H, High dose; and X, extra high dose.

We used network analysis to further explore how increasing the supply of nitrogenous fertilizer altered the genetic capacity for denitrification in the salt marsh plots. We began by examining the role of generalists vs. specialists. Generalist bacteria are able to exploit a diverse array of habitats, though with lower fitness in any given habitat than specialist bacteria (Wilson and Yoshimura, 1994; Kassen and Rainey, 2004). One possible effect of nutrient enrichment could be to increase the abundance of specialist bacteria that are able to thrive under the explicit conditions generated by the nutrient additions. In this experiment we define generalists as those taxa that are present in at least six of the eight marsh plots (green dots, Figure 3A) and specialists as those taxa that are endemic to a single plot (red dots, Figure 3A). Only 11 of the 638 OTUs were present in all eight plots and an additional 20 were found in six or seven of the eight plots.

In contrast to the small number of generalist taxa, when clustered at 88% sequence similarity there were a considerable number of OTUs that were endemic to specific marsh plots (Table 1, Figure 3A). There were between 21 and 140 endemic OTUs, depending on the plot, and these OTUs accounted for between 20 and 53% of all OTUs. Remarkably, even when clustered at only 76% sequence similarity (Figure 3B) 15 of the 27 OTUs were present in only one plot and more then half of these endemic species were found in the XF plots. One XF plot had more endemics than the other when clustered at both 88 and 76% sequence similarity (Figure 3), presumably due to differences in sequencing depth between the two plots (Table 1).

**Figure 4:**
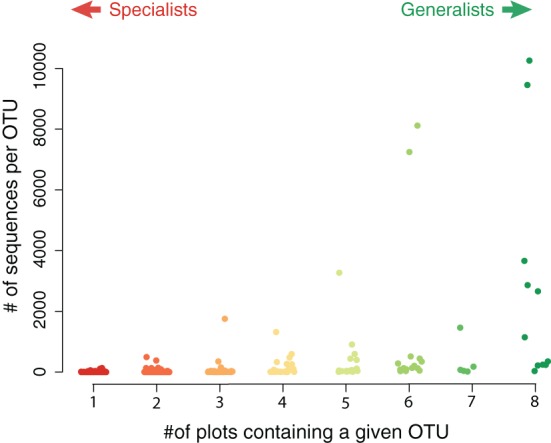
**Abundance of sequences in specialist vs. generalist taxa**. Each value represents the total abundance of an OTU as a function of how many plots contained that sequence. The most abundant sequences were typically present in most, if not all, plots.

Although the endemic OTUs accounted for a large portion of the diversity of the *nirS* gene in the salt marsh plots, they were numerically less abundant than the more generalist taxa (Figure 4). Although not all generalist taxa were highly abundant, the two most abundant OTUs in the marsh were present in all eight plots. Two other highly abundant taxa were present in six of the eight plots (Figure 4) though these two OTUs, notably, were absent from the most highly fertilized plots. By contrast, the taxa that were present in only one or two plots were much lower in abundance than the more ubiquitous sequences. The two most abundant endemic OTUs were present 133 and 110 times in our sequence database and both were found only in plot six, one of the XF plots.

**Figure 5:**
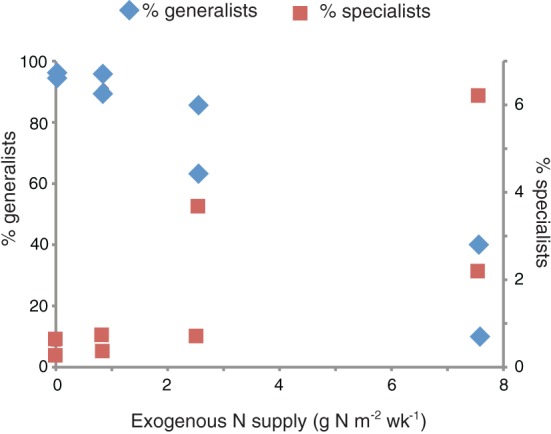
**Relationship between exogenous nitrogen supply and the proportion of generalists (left axis) and specialists (right axis)**. The two symbols per nitrogen supply represent values for the two duplicated plots per treatment.

The proportion of specialists in the marsh plots increased with the amount of exogenous nitrogen added (Figure 5). The 31 generalist OTUs accounted for the vast majority of the sequences in the control and low dose plots (μ = 94.6% ± 2.1). These sequences were also present in the high dose plots, but they accounted for a significantly smaller (Student's *t*-test, *p* = 0.033) proportion of the high dose sequences (μ = 50.0% ± 32.2). The highest number of plot endemics were found in the four most highly fertilized plots (plots 2, 6, 8, and 9).

**Figure 6:**
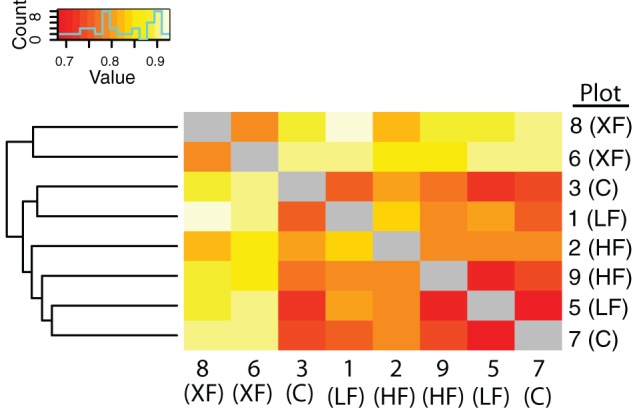
**Heat map of the frequency of co-occurrence of bacterial taxa among the marsh plots**. The less frequent the co-occurrence the warmer the colors. Hierarchical clustering indicates two clusters, one containing the XF plots and the other containing all six remaining plots.

In addition to containing a higher proportion of specialist denitrifiers, the frequency of co-occurrence of *nirS* sequences was lowest among the most highly fertilized plots (Figure 6). Hierarchical clustering of the co-occurrence frequencies identified two clusters, one containing the XF plots, and the other containing the remaining six plots. Within the larger cluster there are two sub-clusters, one containing plots 1 and 2 and the other containing the remaining four plots. Plots one and two are adjacent to one another (Figure 1) and are located a small distance from the remaining plots, therefore this sub-cluster could indicate that dispersal mechanisms are responsible for the patterns of co-occurrence among the remaining plots. The XF plots, however, have the lowest frequency of co-occurrence and do not cluster any where near plot 7, the control plot that is located in between the two XF plots. Thus, dispersal alone cannot explain the co-occurrence patterns observed here.

### Phylogenetic analysis of the *nirS* gene

**Figure 7:**
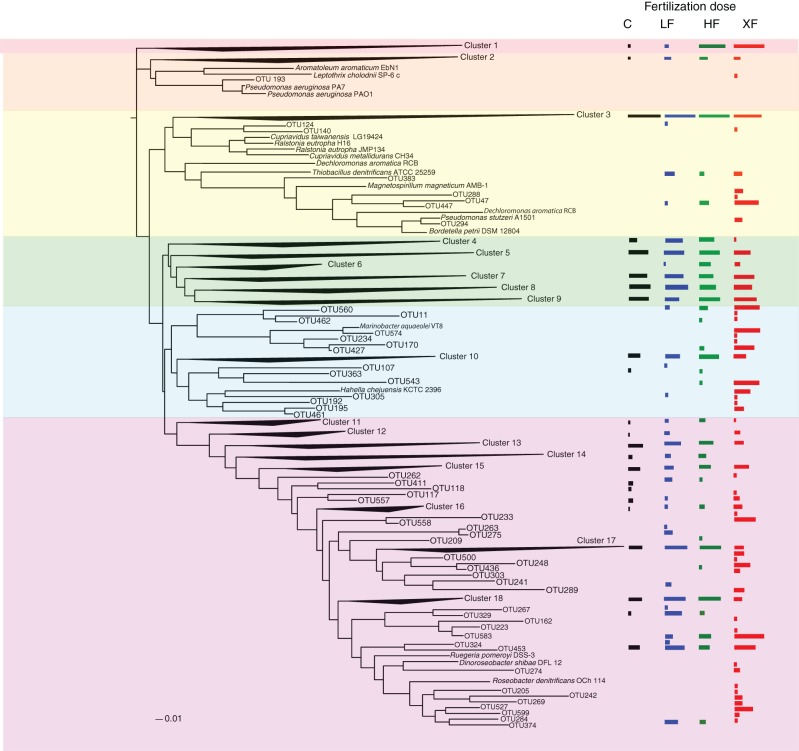
**Phylogenetic tree of the 638 OTUs present in the salt marsh plots**. Where sequences existed that had no close cultured representatives, the sequences were collapsed into clusters. Log normalized relative abundances for each cluster or OTU, plotted as a function of treatment, are displayed on the right of the figure.

The 638 OTUs were divided into six major phylogenetic clades. The first clade (red shading, Figure 7) consisted solely of one cluster of denitrifiers, but among all the clades it showed the strongest taxonomic response to fertilization, with 99.9% of the sequences found in either the HF or XF plots. This clade contained 3142 sequences distributed across 23 OTUs (Table 2). One OTU (634) accounted for the majority of sequences in this clade (Table S3) but it had only a 79% sequence similarity to a cultured representative, *Pseudomonas stutzeri* (Table 3), and a 92% sequence similarity to an environmental clone from the Hai River (accession #: JF966924.1). Clade two (Figure 7, orange shading) contained sequences most closely related to *Pseudomonas aeruginosa*, one of the cultures commonly used to study denitrification. This entire clade, however, consisted of one cluster of low abundance taxa (19 sequences from 13 different OTUs) and one unaffiliated OTU (193), which was a singleton.

**Table 2:** **Summary of the phylogenetic distribution of *nirS* OTUs in 18 clusters represented in six clades on the phylogenetic tree (Figure 8)**.

**Cluster**	**Number**	**Clade**	**C**	**LF**	**HF**	**XF**
	**of OTUs**					
1	23	1	1	2	974	2435
2	13	2	1	5	10	3
3	109	3A	4527	2758	2666	1160
4	26	3B	28	722	451	1
5	18	3B	2789	1819	2829	525
6	10	3B	0	1	100	6
7	69	3B	988	1566	237	2274
8	80	3B	5661	5432	659	702
9	108	3B	1975	266	2758	5580
10	42	3C	138	354	1393	74
11	8	3D	2	2	12	1
12	5	3D	1	0	11	4
13	13	3D	365	543	290	24
14	11	3D	5	7	15	0
15	21	3D	128	36	90	289
16	7	3D	1	3	8	15
17	16	3D	247	4035	4121	19
18	6	3D	2	3	8	15
Unaffiliated	53		19	201	55	2780

For each cluster information is provided on the total number of OTUs per cluster, the representative clade, and the number of sequences found in each of the treatments. Unaffiliated OTUs are those distributed throughout the phylogenetic tree but that did not cluster with any of the other groups.

**Table 3:** **Closest matches of OTUs present more than 1000 times (in descending order from the most abundant) to both a cultured representative and to an environmental clone**.

	**Plot information**	**Closest cultured representative (% match)**	**Closest environmental clone (% match)**
OTU580	All 8 plots	*Azoarcus aromaticum* (78)	Chesapeake Bay (93)
OTU600	All 8 plots	*Brachymonas denitrificans* (82)	Chesapeake Bay (85)
OTU622	Not in XF plots	*Cupriavidus pauculus* (76)	Pearl River Estuary (85)
OTU638	Not in XF plots	*Cupriavidus pauculus* (81)	Bohai Gulf (83)
OTU626	All 8 plots	*Halomonas denitrificans* (73)	Gulf of Mexico Shelf (80)
OTU634	Only in HF and XF	*Pseudomonas stutzeri* (79)	Hai River (92)
OTU629	All 8 plots	*Halomonas nitroreducens* (76)	Hai River (91)
OTU628	All 8 plots	*Pseudomonas aeruginosa* (79)	Bahia del Tobari (89)
OTU625	Abundant in 6 (XF)	*Halomonas koreensis* (80)	Wetland NE Spain (84)
OTU589	Abundant in 6 (XF)	*Dechlorosoma suillum* (86)	California Aquifer (84)
OTU623	Abundant in 6 (XF)	*Halomonas denitrificans* (75)	Chesapeake Bay (81)
OTU627	All 8 plots	*Polymorphum gilvum* (79)	Gulf of Mexico Shelf (79)

Percent values indicate the % match to the associated GenBank sequence. All environmental clones were from sediments of the system listed, except for OTU 589, which was identified in a coastal aquifer.

**Figure 8:**
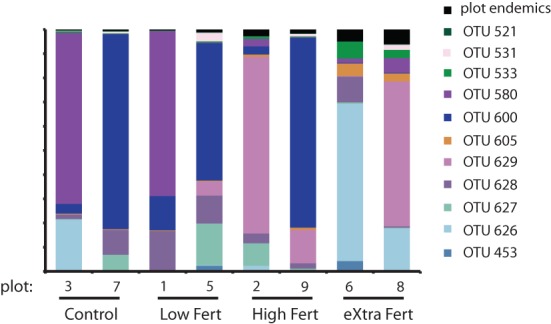
**Stacked bar chart identifying the generalist OTUs that were found in all 8 plots along with the relative proportion of sequences that are endemic to each of the plots**.

The third clade was subdivided into four groups (3A–3D). Clade 3A (Figure 7, yellow shading) contained one of the most abundant clusters of *nirS* denitrifiers. Cluster three (Table 2) contained 11,111 sequences from 109 OTUs. The control plots had the largest number of sequences in this clade. Three sequences from clade 3A (OTUs 521, 600, and 605) were present in all eight plots (Figure 8), and OTU600 was the second most abundant sequence overall. This OTU was an 82% match to the beta-proteobacteria *Brachymonas denitrificans* (Table 3), and an 85% match to an environmental clone from the Chesapeake Bay (accession #: DQ676092.1). Although abundant in plots 5 (LF), 7 (control), and 9 (HF), it was completely absent from the XF plots. Clade 3B (Figure 7, green shading) consisted of 6 clusters of *nirS* denitrifiers and accounted for more than half of all the sequences discovered, yet none of these clusters had close cultured representatives. Within this clade, cluster 8 had many more sequences in the control and low dose plots than in the highly fertilized plots, while cluster 9 showed the reverse pattern, with much greater sequence abundance in the highly fertilized plots than in the control and low dose plots (Table 2). Cluster 8 contained the most abundant OTU identified in the marsh plots (OTU580; Figure 8), whose closest cultured representative, the beta-proteobacteria *Azoarcus aromaticum* (Table 3), shared only 78% sequence similarity. This OTU shared a 93% sequence identity to the same Chesapeake Bay environmental clone identified above. This OTU, however, was found in high abundance only in plot 1 (LF) and plot 3 (control). Clade 3C (Figure 7, blue shading) contained one cluster of 42 OTUs, the majority of which were found in the HF plots (Table 2), as well as some unaffiliated OTUs that clustered together with *Marinobacter* and *Hahella* species. This clade also had a number of taxa that were present only in the XF plots. The last clade, 3D (Figure 7, violet shading) contained several smaller clusters as well as many unaffiliated OTUs. Cluster 17 contained the most sequences (8422) in clade 3D, 99% of which were found in the LF and HF plots (Table 2).

## Discussion

### Using AIC scores to assess models for sequence clustering

Finding a meaningful definition of “species” among bacteria has been a subject of considerable debate (Cohan, 2002; Stackebrandt et al., 2002; Gevers et al., 2005; Konstantinidis et al., 2006). Based on genetic analysis compared with DNA-DNA hybridization techniques, it is generally accepted that different bacterial OTUs are defined at less than 97% sequence similarity in the 16s rRNA gene (Gevers et al., 2005), and multiple different algorithms (summarized in Lozupone and Knight, 2008) have been developed to analyze community structure based on the 16S rRNA derived phylogenies. These algorithms, and the 97% clustering threshold, do not implicitly apply to protein coding genes. Different protein coding genes will accrue mutations at different rates depending on specific evolutionary pressures so defining a universal degree of clustering to operationally define bacterial taxonomic units using protein coding genes is inappropriate. Nonetheless, for any given protein coding gene, we need useful mechanisms to define ecologically relevant OTUs that are complementary to methods already developed for the analysis of 16S rRNA genes.

We modified an approach used in food web ecology wherein the best model for trophic structure is identified using AIC (Allesina and Pascual, 2009; Baskerville et al., 2011). Rather than attempting to delineate trophic structure, however, we applied the same principle to derive a novel technique to define the most ecologically relevant OTUs for the study of *nirS* genes in the environment. Furthermore, we built on the framework of Allesina and Pascual to incorporate abundance information using weighted networks. This technique should be useful for food webs and other weighted ecological networks as well. By integrating sequence abundance information within an ecological network of plots we were able to determine the degree of sequence clustering that resulted in the best structure of the network (as defined by the network formulation when no clustering was used) when the loss of information achieved by clustering was taken into account. The results indicated that through a range of sequence clustering (82–97% similarity, Figure 2) very similar AIC scores were achieved, suggesting that conclusions about community structure drawn from *nirS* genes may be robust to the degree of sequence clustering. The sharp increase in AIC scores when sequences were clustered at 80% sequence similarity and below suggests that there is a clear point at which relevant ecological information is lost. Whether or not the threshold of 88% sequence similarity applies to other functional genes remains to be tested, but this approach provides one avenue for beginning to assess the appropriate degree of sequence clustering for protein coding genes.

### The role of increased nitrogen supply in structuring the denitrifying bacterial community

The role that exogenous nutrients play in structuring microbial systems has received considerable attention, but the results of different studies lead to equivocal conclusions. A recent meta-analysis indicated that 84% of studies showed some sensitivity by microbes to nutrient enrichment (Allison and Martiny, 2008). These results have since been supported by additional research that showed shifts in soil microbial communities as a result of increased nutrient supply (Fierer et al., 2012). In salt marshes, however, microbes appeared to be resistant to long-term nutrient enrichment in two different marsh systems (Bowen et al., 2009, 2011).

The response of specific functional genes to nutrient enrichment has also led to ambiguous results. Soil microbial communities have shown strong response by protein coding genes to nutrient enrichment (Enwall et al., 2005, 2007; Fierer et al., 2012). By contrast, in salt marsh sediments, analysis of the ammonia monooxygenase (*amoA*) gene in both ammonia oxidizing archaea and bacteria indicated a fertilization induced shift in bacterial *amoA* but not in archaeal *amoA* (Peng et al., 2012). There was no significant shift in the structure of the nitrogen fixing bacterial community as determined by analysis of the nitrogenase gene in marsh sediments (Piceno and Lovell, 2000; Lovell et al., 2001), and there was no difference in the community structure of denitrifying bacteria as a result of nutrient enrichment when examined using a functional gene microarray (Bowen et al., 2011).

The in depth examination of the community structure of denitrifiers from marsh plots that we report provides evidence that there are, in fact, changes to the distribution of the *nirS* functional gene as a result of fertilization. Our data indicate that fertilization increases both the number of singletons found in marsh plots as well as the number of taxa that are endemic to a specific plot. Several taxa were present in high abundances only in the HF or XF plots and may indicate taxa that are able to specialize on the specific conditions induced by the fertilization. These conclusions would suggest that increasing the supply of nutrients may provide additional niche space where specialist bacteria are able to thrive at the expense of the handful of generalist taxa that dominate the sequence abundances of the control and low dose plots. Even when clustered at very low degrees of sequence similarity (76%; Figure 3B) the highly fertilized plots showed a large degree of taxonomic endemism, suggesting that the supply of anthropogenic nitrogen may promote the success of unique denitrifiers with highly divergent nirS sequences. The role that these rare and unique denitrifiers play in the biogeochemistry of the plots requires further exploration, but their greater abundance in the highly fertilized marsh plots suggests that the overall genetic capacity for denitrification may be enhanced as a result of the nutrient additions.

Although singeltons could be a result of sequencing errors that escaped detection by our quality control measures, we have previously shown that the likelihood of this is rare. Our functional gene analysis pipeline had an error rate of 0.0–0.18% when used to analyze pyrosequencing data of known controls (Weisman et al., 2013), furthermore, this pipeline removes more spurious diversity while retaining a greater number of real *nirS* sequences than approaches based solely on the removal of sequences with unexpected stop codons (Weisman et al., 2013). Finally, the realization that the number of singletons is not evenly distributed over samples from all the plots provides further evidence that this result is not likely a product of random sequencing error. Additionally, it is possible that low abundance taxa that we interpret to be specialists could, in fact, be generalists that were not detected due to incomplete sampling. Such methodological limitations have been suggested previously (Barberán et al., 2012) and cannot be discounted here. However, if this were the case here, then there should be a correlation between the number of individuals sequenced per plot and the number of endemics or singletons identified in the plot (Table 1). No such correlation exists among these samples (*R*^2^ < 0.1). Furthermore, as we change the degree of clustering used to define OTUs from 88 to 76% (Figure 3, Table S1), we still see a greater number of specialist taxa in the highly fertilized plots. If this were solely a function of incomplete sequencing the presence of specialist taxa should be randomly distributed across the plots. Taken together, these points provide some confidence that this is a biologically relevant distribution, rather than an artifact of incomplete sampling.

Although the data analysis pipeline we employed (Weisman et al., 2013) removes spurious sequences at rates consistent with those designed to remove sequencing artifacts from 16S rRNA data (Huse et al., 2010), there are additional sources of bias that suggest we are underestimating the overall genetic capacity for denitrification. First, our study only targets the *nirS* gene-containing denitrifiers. Denitrifiers that contain the functionally equivalent gene *nirK* have been documented in marsh sediments (Beazley et al., 2012) and could be contributing to denitrification rates in these sediments, but their contribution was not assessed in this study. Previous work, however, suggests that in marine systems the contribution of *nirK*-based denitrifiers is small, though perhaps underreported (Jones and Hallin, 2010). Second, additional bias could result from differential amplification of sequences in the multi-template PCR (Polz and Cavanaugh, 1998), an effect that could also be increased by the nested nature of the PCR (Pinto and Raskin, 2012). These types of biases are typically manifested in pyrosequencing data by altering metrics based on either the rank abundance of taxa or on richness estimators derived from the number of low abundance taxa (Pinto and Raskin, 2012). The network analyses used in this work, however, avoid the biases associated with these metrics, which is one of the major strengths of network-based approaches. Third, we may be underestimating the total genetic capacity for denitrification because any *nirS* containing microbes whose DNA sequences are sufficiently divergent in the priming region that they cannot be amplified by these primers will not be detected.

The data presented here, suggesting that fertilization does alter the structure of the microbial community, perhaps by enhancing the niche space for bacterial specialists, contradicts early work of ours that indicated very little change in the microbial community as a result of fertilization (Bowen et al., 2011). In that analysis the overall bacterial community composition was assessed via pyrosequencing of the 16S rRNA gene, similar to the methods used in this study to analyze the *nirS* gene. In our previous *nirS* work, however, we used an early generation functional gene microarray to assess how denitrifiers responded to increased nitrogen supply. This early generation microarray contained 39 *nirS* oligonucleotide probes derived from sequences that existed in public databases at the time of array development (Bulow et al., 2008). A comparison between the sequence data derived from this study and the oligonucleotides present on the array indicate, however, that the *nirS* sequences that we identified in the marsh are considerably different from those present in public databases (Table 3) and the majority of these sequences, even the most abundant ones, would have escaped detection by the microarray. We conclude that pyrosequencing of functional gene amplicons provides a much more nuanced examination of microbial community structure than is possible with microarrays unless the underlying genetic structure of the system in which the microarray is being employed is incorporated into the microarray probe set.

Many pyrosequencing papers have demonstrated that microbial communities consist of a few very abundant taxa and a large number of taxa that are present in low abundance (Sogin et al., 2006; Galand et al., 2009; Brazelton et al., 2010). It appears that, at least for taxa containing the *nirS* gene, this pattern is consistent. The role that these low abundance *nirS* OTUs play in the ecology of the salt marsh plots requires further investigation, however recent examinations of the 16S rRNA:rDNA ratios in coastal ocean water indicate that the active portion of the microbial community is often overrepresented among the less abundant taxa (Campbell et al., 2011; Campbell and Kirchman, 2012). Further assessment of the active portion of the denitrifying community in the marsh plots is needed to ascertain what role these rare denitrifiers play in the biogeochemistry of the salt marsh.

Salt marshes exist at a critical interface between land and sea and they play an important role in protecting coastal waters from land-derived nutrient pollution (Valiela and Cole, 2002). Marsh sediments support some of the highest rates of denitrification measured (Hopkinson and Giblin, 2008) and rates of denitrification appear to be enhanced by increased nutrient supply both in these marsh plots (Hamersley and Howes, 2005) and in other studies (Koop-Jakobsen and Giblin, 2010; Vieillard and Fulweiler, 2012). Here, we present the first evidence that increasing the supply of nutrients to marsh sediments also changes the structure of the denitrifying community by increasing the proportion of bacterial specialists and decreasing the abundance of generalist taxa in plots receiving increased nutrients.

Our data also indicated that the denitrifiers that are present in the marsh are not at all similar to those that currently exist in culture, underscoring the importance of continuing to isolate novel bacteria from a wide variety of environments so that explicit metabolic pathways can be investigated. Overall the highly abundant taxa were only distantly related (μ = 76.7 ± 3.5% sequence similarity) to any cultured representatives (Table 3). These sequences were more closely related to sequences derived from environmental clones (μ = 85.5 ± 4.7% sequence similarity). In most cases these clones were derived from coastal and estuarine sediments throughout the world (Table 3). Future work is needed to (1) determine the metabolic pathways employed by these denitrifiers, (2) establish what proportion of the denitrifiers are active, and most importantly (3) link the active portion of the community with measured rates of denitrification to further elucidate the connection between marsh microbial diversity and geochemical function.

## Author contributions

Jennifer L. Bowen designed the research, performed all sample preparation, and oversaw the DNA amplification and sequencing. Jarrett E. K. Byrnes, conceived of and performed the AIC analysis and the network analysis. David Weisman developed the bioinformatics pipeline for analyzing the high throughput DNA sequences. Cori Colaneri performed the phylogenetic analysis. All authors contributed to the writing of the manuscript.

### Conflict of interest statement

The authors declare that the research was conducted in the absence of any commercial or financial relationships that could be construed as a potential conflict of interest.

## Supplementary material

The Supplementary Material for this article can be found online at:

www.frontiersin.org/Journal/10.3389/fmicb.2013.00342/abstract
