# Supplementary material for: Functional gene pyrosequencing and network analysis: an approach to examine the response of denitrifying bacteria to increased nitrogen supply in salt marsh sediments
Source: Front Microbiol. 2013 Nov 27;4:342. doi: 10.3389/fmicb.2013.00342 (PMC3841915; doi:10.3389/fmicb.2013.00342)
Supplement: Supplementary file 1 [file Presentation1.PDF]

Supplemental Figures

Figure S1: Bipartite graph of the distribution of operational taxonomic units (OTUs) in each of the plots for OTUs defined at 99% sequence similarity (A) and 74% sequence similarity (B). Plots are displayed on the lower X axis and OTU space on the upper X axis. The weight of the line indicates the relative abundance of sequences in each OTU.

Figure S2: Bipartite graph of the distribution of operational taxonomic units (OTUs) in each of the plots when OTUs are defined at greater than 88% sequence similarity. Plots are displayed on the lower x-axis and OTU space on the upper x-axis. The weight of the line indicates the relative abundance of sequences in each OTU.

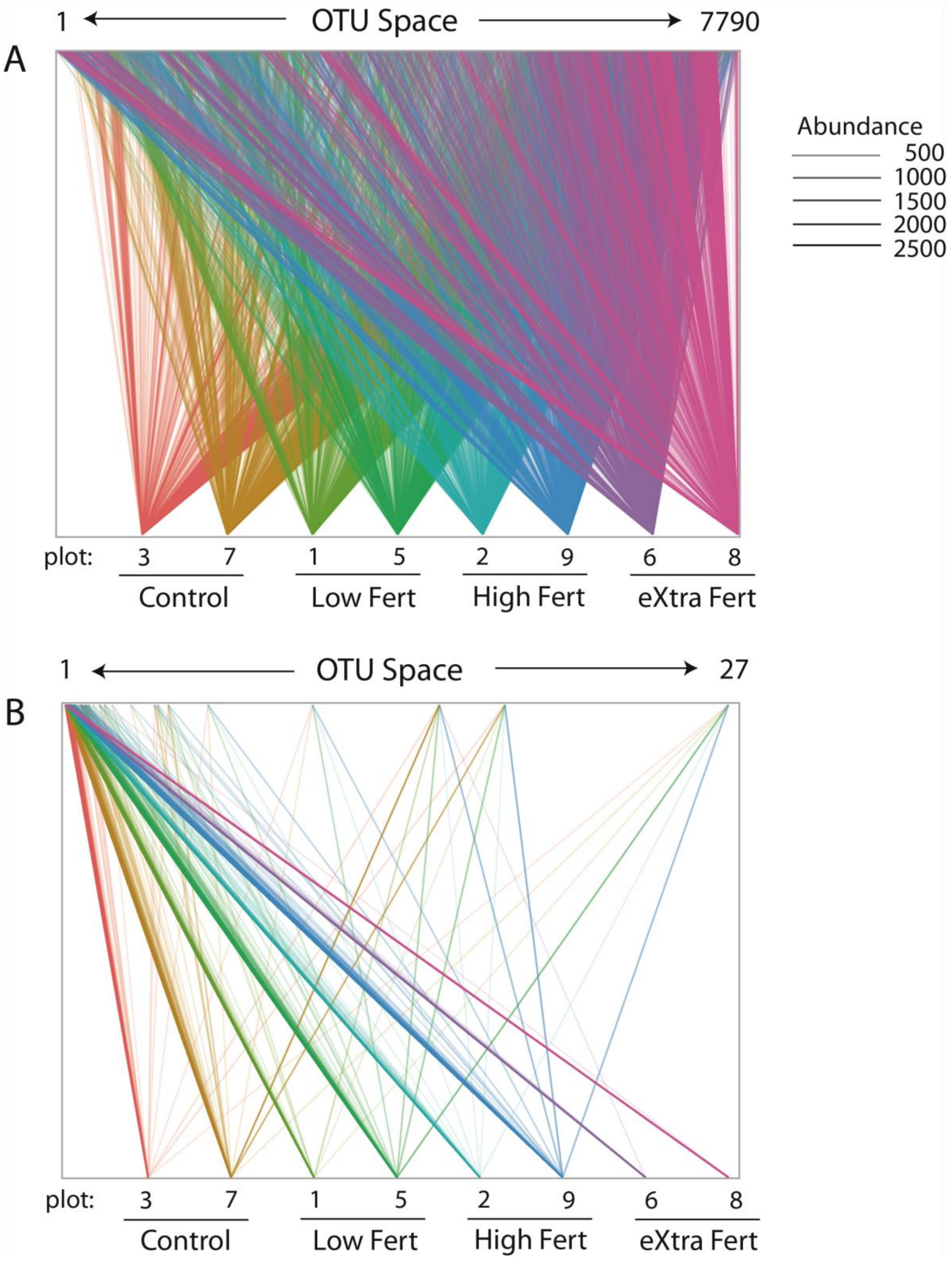

1
OTU Space
7790
A
Abundance
500
1000
1500
2000
2500
plot:
3
7
1
5
2
9
6
8
Control
Low Fert
High Fert
eXtra Fert
1
OTU Space
27
B
plot:
3
7
1
5
2
9
6
8
Control
Low Fert
High Fert
eXtra Fert

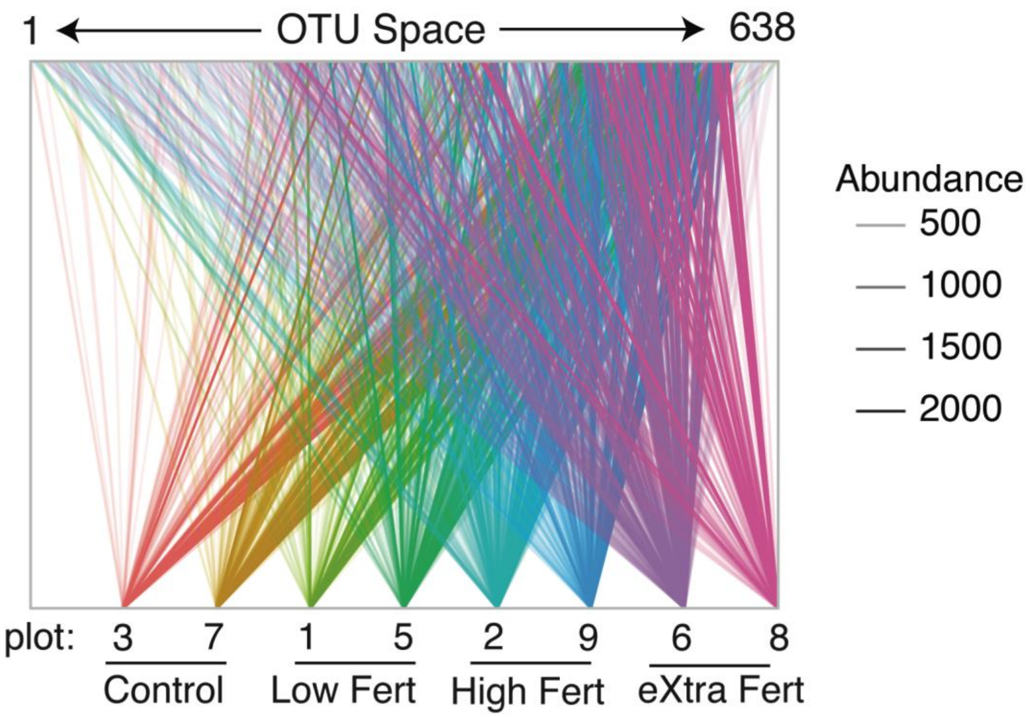

1
OTU Space
638
Abundance
500
1000
1500
2000
plot:
3
7
1
5
2
9
6
8
Control
Low Fert
High Fert
eXtra Fert
