# Supplementary material for: Functional gene pyrosequencing and network analysis: an approach to examine the response of denitrifying bacteria to increased nitrogen supply in salt marsh sediments
Source: Front Microbiol. 2013 Nov 27;4:342. doi: 10.3389/fmicb.2013.00342 (PMC3841915; doi:10.3389/fmicb.2013.00342)
Supplement: Supplementary file 3 [file DataSheet2.DOCX]

Table S2: AIC scores calculated for each sequence cluster generated. Clustering at 88% sequence identity produced the lowest AIC score.

| Sequence cluster (%) | AIC score |
| --- | --- |
| 99 | 664,030 |
| 98 | 625,588 |
| 97 | 609,301 |
| 96 | 602,568 |
| 95 | 601,034 |
| 94 | 596,703 |
| 93 | 592,612 |
| 92 | 589,033 |
| 91 | 588,141 |
| 90 | 588,087 |
| 88 | 587,792 |
| 86 | 592,150 |
| 84 | 597,204 |
| 82 | 610,916 |
| 80 | 767,696 |
| 78 | 763,529 |
| 76 | 804,163 |
| 74 | 805,140 |
